# Renal resistive index can predict response to furosemide in septic patients with oliguria

**DOI:** 10.1186/cc12358

**Published:** 2013-03-19

**Authors:** I Gornik, A Godan, V Gasparovic

**Affiliations:** 1 University Hospital Centre Zagreb, Croatia

## Introduction

Oliguria is common in septic patients and is frequently therapeutically addressed with loop diuretics; that is, furosemide. Diuretic treatment in shock and hypovolemia is not rational, but can be tried in oliguric patients with normovolemia or hypervolemia and without hypotension. In such patients it still does not always increase dieresis and can also be harmful. The resistive index is a measure of pulsatile blood flow that reflects the resistance to blood flow caused by the microvascular bed distal to the site of measurement. It can reflect functional status of the tissue distal to the point of measurement. We investigated whether measuring the renal resistive index (RI) could be helpful in determining which patients will respond to furosemide treatment.

## Methods

We included medical ICU patients with sepsis and oliguria (urine output <1 ml/kg/hour) who were prescribed i.v. furosemide. Patients with known chronic renal failure, hypovolemia (CVP <10 mmHg) or severe hypotension (MAP <80 mmHg) were excluded. Resistive index (1 - (end diastolic velocity/maximum systolic velocity)×100) was measured in at least three segmental arteries of both kidneys, the average of all measurements was reported as the result. Repeated assessments were viewed as independent if separated by more than 24 hours. Furosemide was given intravenously in the dose of 40 mg after RI measurement. Positive response to furosemide was defined as doubling of hourly dieresis or achieving urine output >1.5 ml/kg/hour after drug administration.

## Results

We included 47 patients with a total of 59 measurements. In 28 cases patients had positive response to furosemide. Median RI in responders was 0.67 (range 0.55 to 0.78) and in nonresponders 0.79 (range 0.58 to 0.81); *P *= 0.027. Construction of receiver operating characteristic curve showed 83% sensitivity and 81% specificity for the cutoff RI 0.73. No other measured patient characteristic was found to be predictive of response to diuretic treatment.

## Conclusion

Our results show that the RI could be used to guide diuretic treatment in nonhypovolemic, nonhypotensive septic patients. Further studies are needed to confirm those preliminary results.

